# Evaluation of rheological properties of soft lining materials with different composition under various temperatures

**DOI:** 10.1186/s12903-024-04393-5

**Published:** 2024-05-28

**Authors:** Şule Nur Macit, Ayhan Gürbüz, Perihan Oyar

**Affiliations:** 1 Ministry of Health, Kırıkkale Oral and Dental Health Center, Ankara, Turkey; 2 Department of Prosthodontics, Faculty of Dentistry, Ankara University, Ankara, Turkey; 3 Department of Dental Prostheses Technology, Vocational School of Health Services, Hacettepe University, D Block, 3. Floor, Sihhiye, 06100 Ankara Turkey

**Keywords:** Rheology, Rheometer, Soft denture liner, Temperature effect, Viscoelasticity

## Abstract

**Purpose:**

The aim of this in vitro study was to evaluate the changes the rheological properties of some soft lining materials, to compare the rheological properties and viscoelastic behaviour at different temperatures.

**Materials and methods:**

Five soft lining materials (acrylic and silicone based) were used. the storage modulus (G’), loss modulus (G”), tan delta (tan δ) and complex viscosity (η’) were chosen and for each material, measurements were repeated at 23, 33 and 37  °C, using an oscillating rheometer. All data were statistically analyzed using the Mann Whitney U test, Kruskal Wallis test and Conover’s Multiple Comparison test at the significance level of 0.05.

**Results:**

Soft lining materials had different viscoelastic properties and most of the materials showed different rheological behavior at 23, 33 and 37  °C. At the end of the test (t¹5), at all the temperatures, Sofreliner Tough M had the highest storage modulus values while Visco Gel had the highest loss Tan delta values.

**Conclusions:**

There were significant changes in the rheological parameters of all the materials. Also temperature affected the initial rheological properties, and polymerization reaction of all the materials, depending on temperature increase.

**Clinical implications:**

Temperature affected the initial rheological properties, and polymerization reaction of soft denture liner materials, and clinical inferences should be drawn from such studies conducted. It can be recommended to utilize viscoelastic acrylic-based temporary soft lining materials with lower storage modulus, higher tan delta value, and high viscosity in situations where pain complaint persists and tissue stress is extremely significant, provided that they are replaced often.

## Background

Especially for weak elderly patients with fragile health who cannot endure the hard acrylic denture foundation, soft lining materials are employed to replace the inner surface of a standard complete denture [1]. These soft lining materials have different viscoelastic properties. Viscosity and flow, which vary depending on the product or the environment, are rheological variables that have a big impact on handling characteristics [2, 3]. The use of rheology in the dental field is very important in terms of its contribution to new dental material formulations and the development of the use properties of existing materials, increasing their long-term effectiveness [4–6]. The durability and viscoelastic qualities of these soft lining materials, which describe the material’s capacity to produce the cushioning effect, are thought to have the biggest effects on their efficacy [7, 8]. Numerous investigations looking into the viscoelastic characteristics of these soft lining materials have suggested that the polymerization reaction of elastomeric materials is very sensitive to changes in temperature and considerably impacts rheological parameters (2, 9–15). Data from earlier papers further emphasize how crucial it is to look into the curing reaction’s relationship to temperature in more detail [2, 9–13]. For this reason, the purpose of this in vitro study was to compare the rheological characteristics and viscoelastic behavior at various temperatures, investigate changes in the rheological properties of some soft lining materials that have recently become popular, and gather information that might help in the selection process.

The null hypothesis was that there was no difference among soft lining materials for the storage modulus (G’) and the loss tan delta (tan δ) at 2 times (t0 and t¹5 and at 3 temperatures (23, 33 and 37 °C).

## Materials and methods

**Fig. 1:**
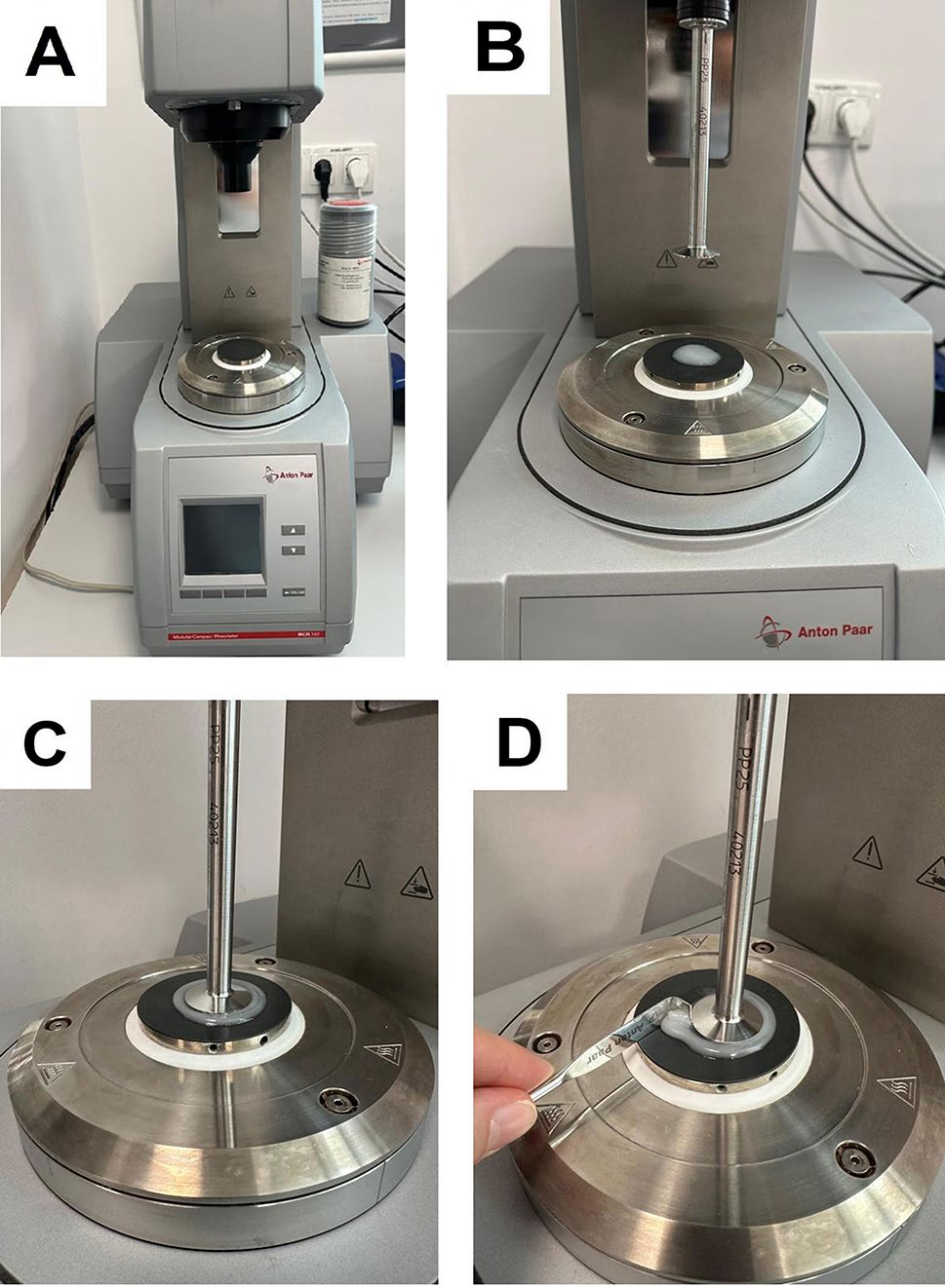
**A**: Peltier device, **B**: Material on the lower plate, **C**: Compression of material between plates, **D**: Removal of extra material

In this study, five soft lining materials (n:11) with acrylic temporary materials (Visco Gel, Trusoft) and silicone permanent soft lining materials (Sofreliner Tough S, Sofreliner Tough M, Ufi Gel P) were investigated. The tested specimens have autopolymerization and the reaction of entanglement system. The materials are listed in Table 1. The manufacturer’s instructions (Table 1) were followed for preparation of each specimen. Oscillatory rheometer (Anton Paar MCR 102; Anton Paar GmbH) was used for examining the rheological properties. The storage modulus (G’), loss modulus (G”), tan delta (tan δ) and complex viscosity (η’) were chosen to represent the rheological properties of the materials until the time of trimming. A 25 mm diameter parallel plate was used in oscillatory rheometer with isothermal curing, gelafication test mode at 1 Hz frequency and the gap width was calibrated at 1000 µm [12]. After the specimens were prepared according to the manufacturer’s directives (Table 1), they were immediately placed on the plate of the rheometer. A Peltier device kept the specimen at the desired temperature on the lower plate. The top plate was lowered into place after the bottom plate (Peltier) had been lifted by 5 cm, the specimens had been placed directly on it, the extra material had been removed from the gap’s outside edge, and the measurement was started as the upper plate was set into oscillation. 40 seconds (marked as to) after the specimens were placed on the plate, the measurements began (Fig. 1). Datas were obtained every 30 second for total time period of 15 minute (designated as t¹5) and monitored using device-specific software (Anton Paar RheoCompass 1.18; Anton Paar GmbH). For all of the materials, measurements were repeated at 23, 33 and 37°C. Thus, a total of 165 measurements were obtained. The storage modulus, loss modulus, tan delta, and complicated viscosity measurements made while maintaining a constant material and temperature were compared between the follow-up times using the Friedman (non parametric) test to determine whether there was a statistically significant difference. The Wilcoxon Sign test (non parametric) was used to identify the follow-up time(s) that contributed to the difference in these instances based on the significant Friedman test data. When there were two independent groups, the Mann Whitney U test (non parametric) was used to determine the significance of the measurement difference between materials used in various structures, and the Kruskal Wallis test (non parametric) was used to determine the significance of the measurement difference between more than two groups. Conover’s Multiple Comparison test was employed to determine the situation(s) producing the difference in cases where the results of the Kruskal Wallis test statistic were determined to be significant. Results for P < .05, were considered statistically significant. In all possible multiple comparisons, Bonferroni Correction was performed to control the Type I error.

**Table 1:** Materials

Material	Group	Manufactures [Preparation]	Generic types	Product types	Batch no
Visco Gel	A	Dentisply [1 Measure powder (3 g) and 1 measure liquid (2.2 ml)]	Plasticized PMMA/PEMA	Powder/Liquid	1,608,000,400
Sofreliner Tough S	B	Tokuyama Corp. [Mix paste for 1.5–2 min and wait 20 min]	Poly(siloxane)	Paste/Paste	036E57
Sofreliner Tough M	C	Tokuyama Corp. [Mix paste for 1.5–2 min and wait 20 min]	Poly(siloxane)	Paste/Paste	038E57
Ufi Gel P	D	VOCO GmbH [Mix base and catalyst in 1:1 ratio within 30 s]	Poly(siloxane)	Paste/Paste	1,648,489
Trusoft	E	Bosworth Company [Mix in 1:1 ratio within 30 s, let stand for 15 s and wait 1–2 min]	Plasticized PMMA/PEMA	Powder/Liquid	FL1240

## Results

**Fig. 2:**
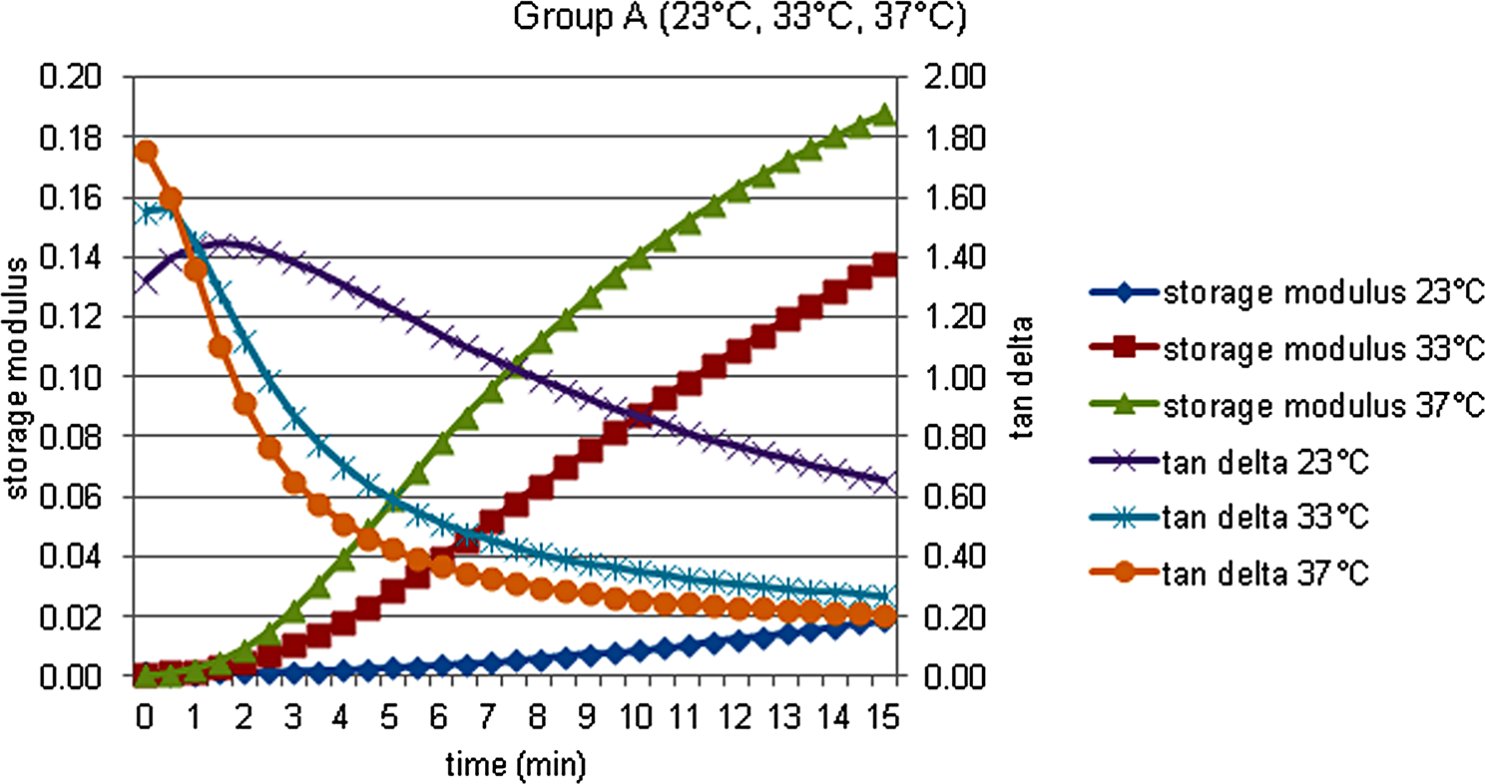
Storage modulus and tan delta against time measured at 23, 33, and 37  °C in Group A

**Fig. 3:**
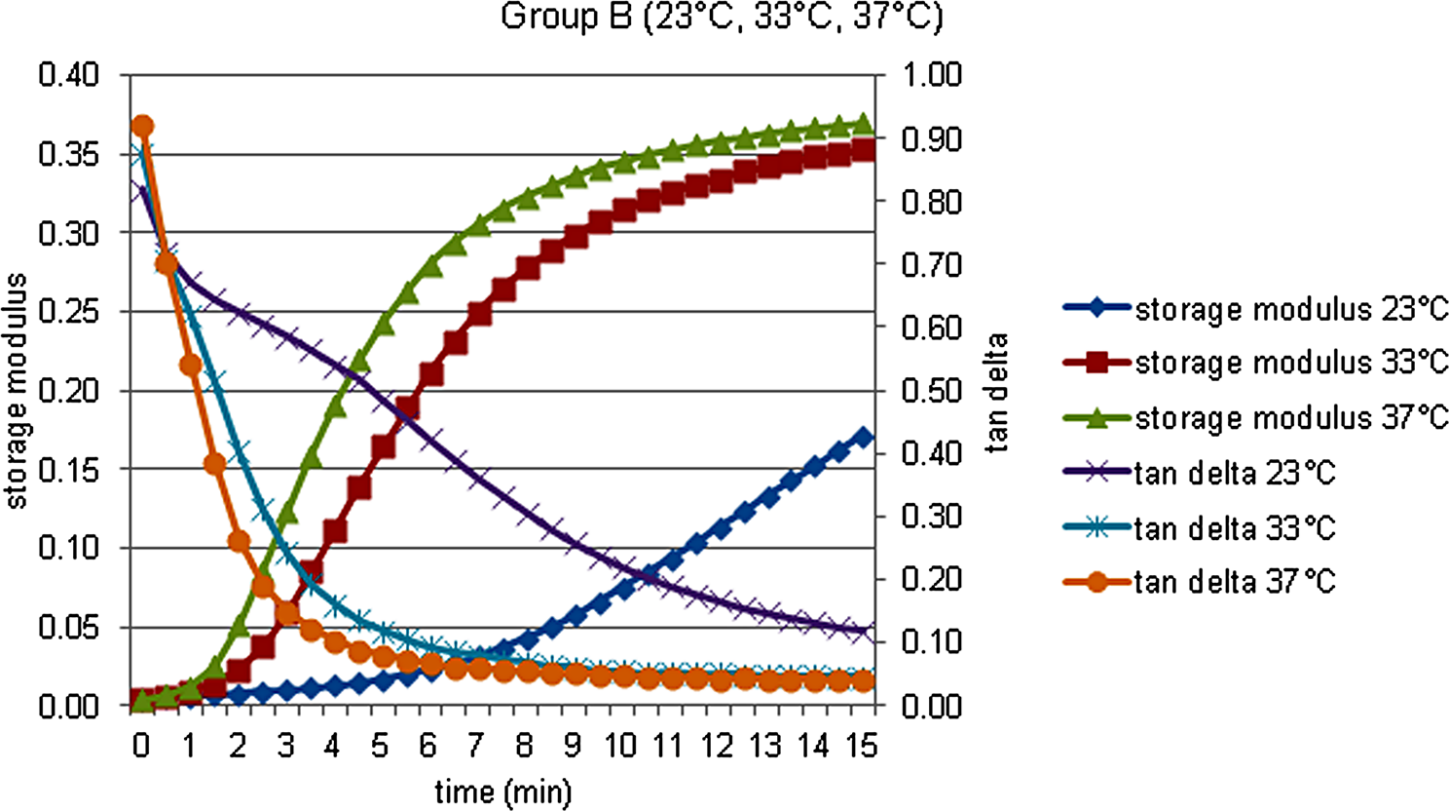
Storage modulus and tan delta against time measured at 23, 33, and 37  °C in Group B

**Fig. 4:**
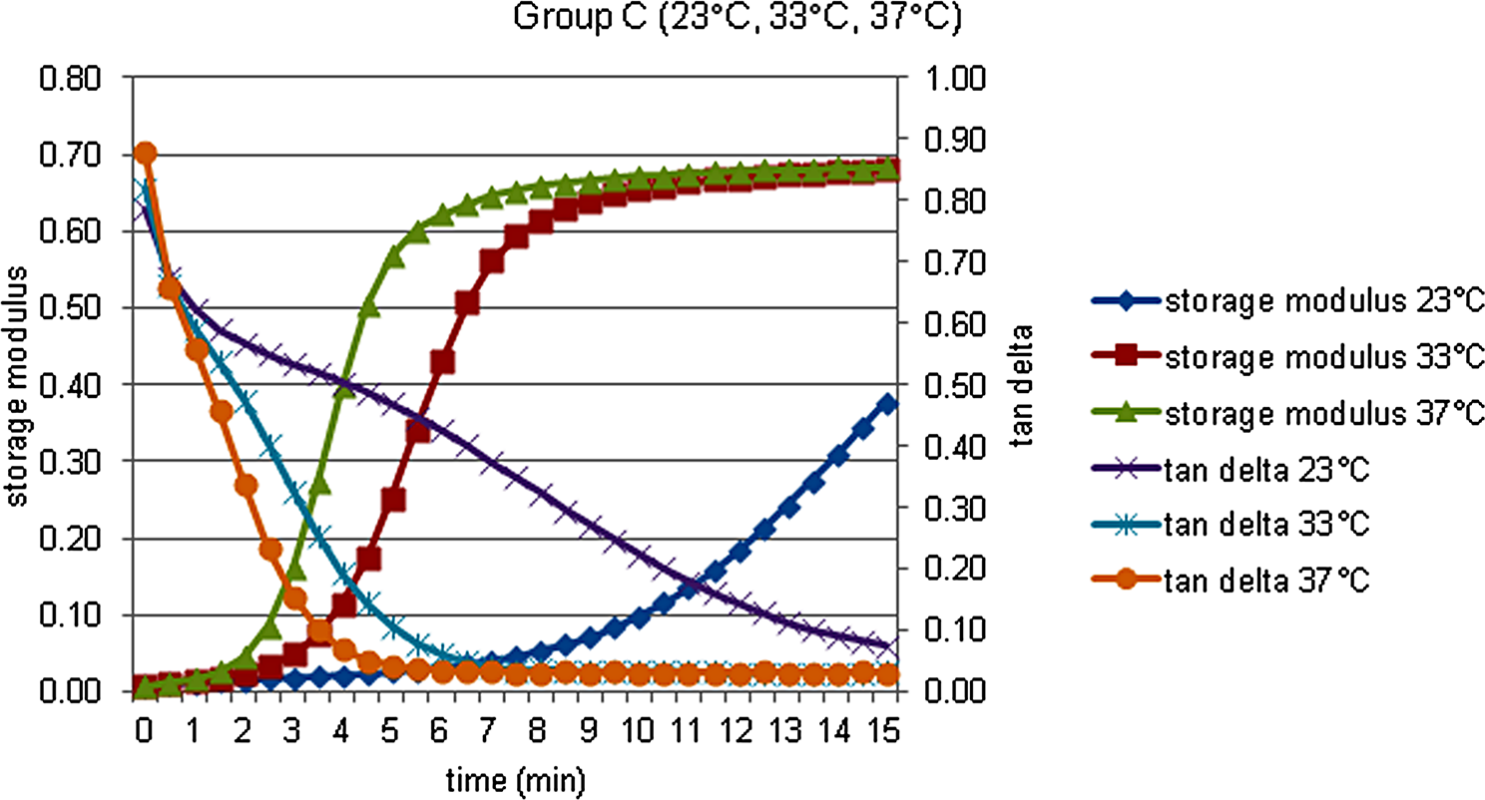
Storage modulus and tan delta against time measured at 23, 33, and 37  °C in Group C

**Fig. 5:**
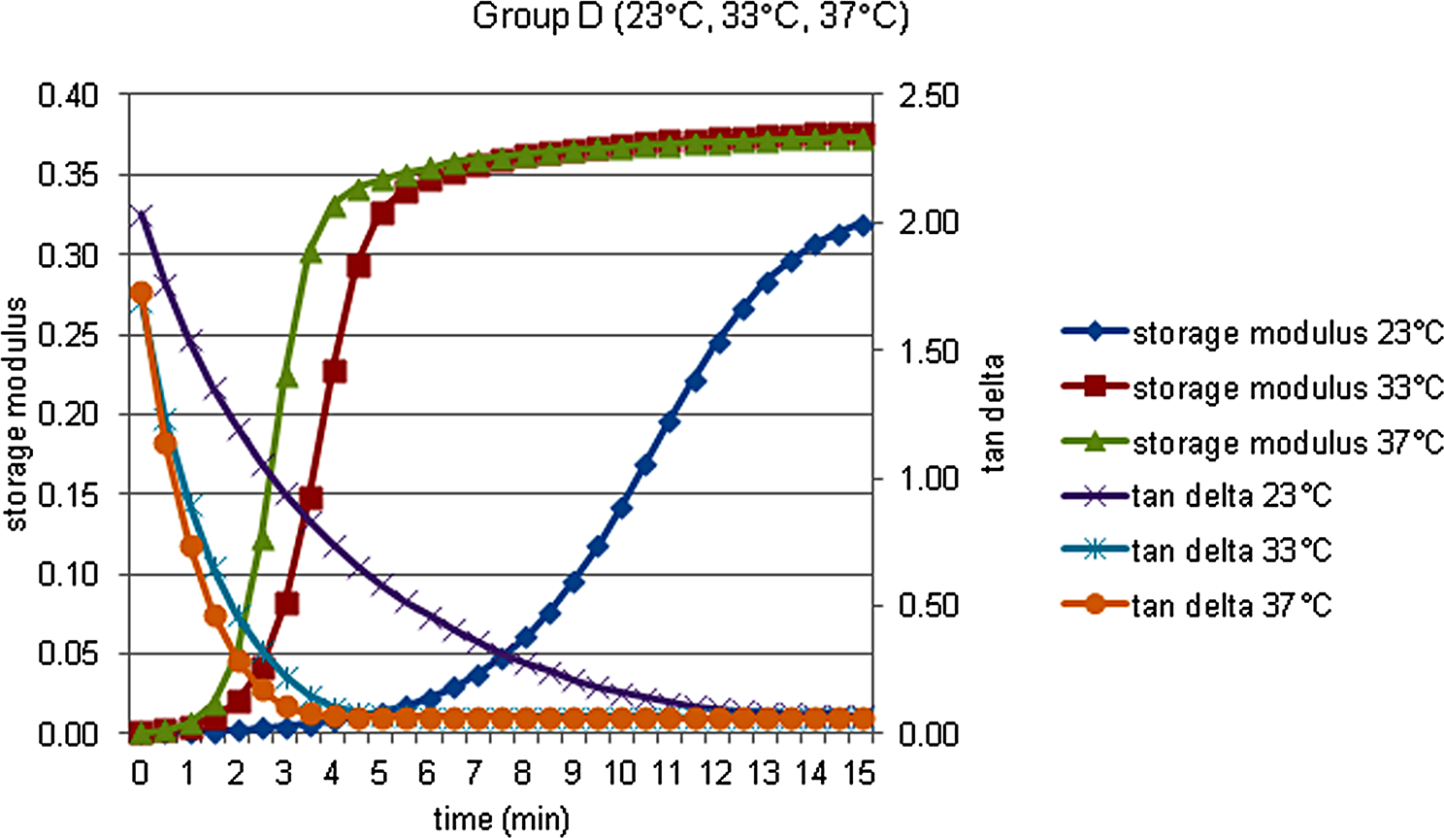
Storage modulus and tan delta against time measured at 23, 33, and 37  °C in Group D

**Fig. 6:**
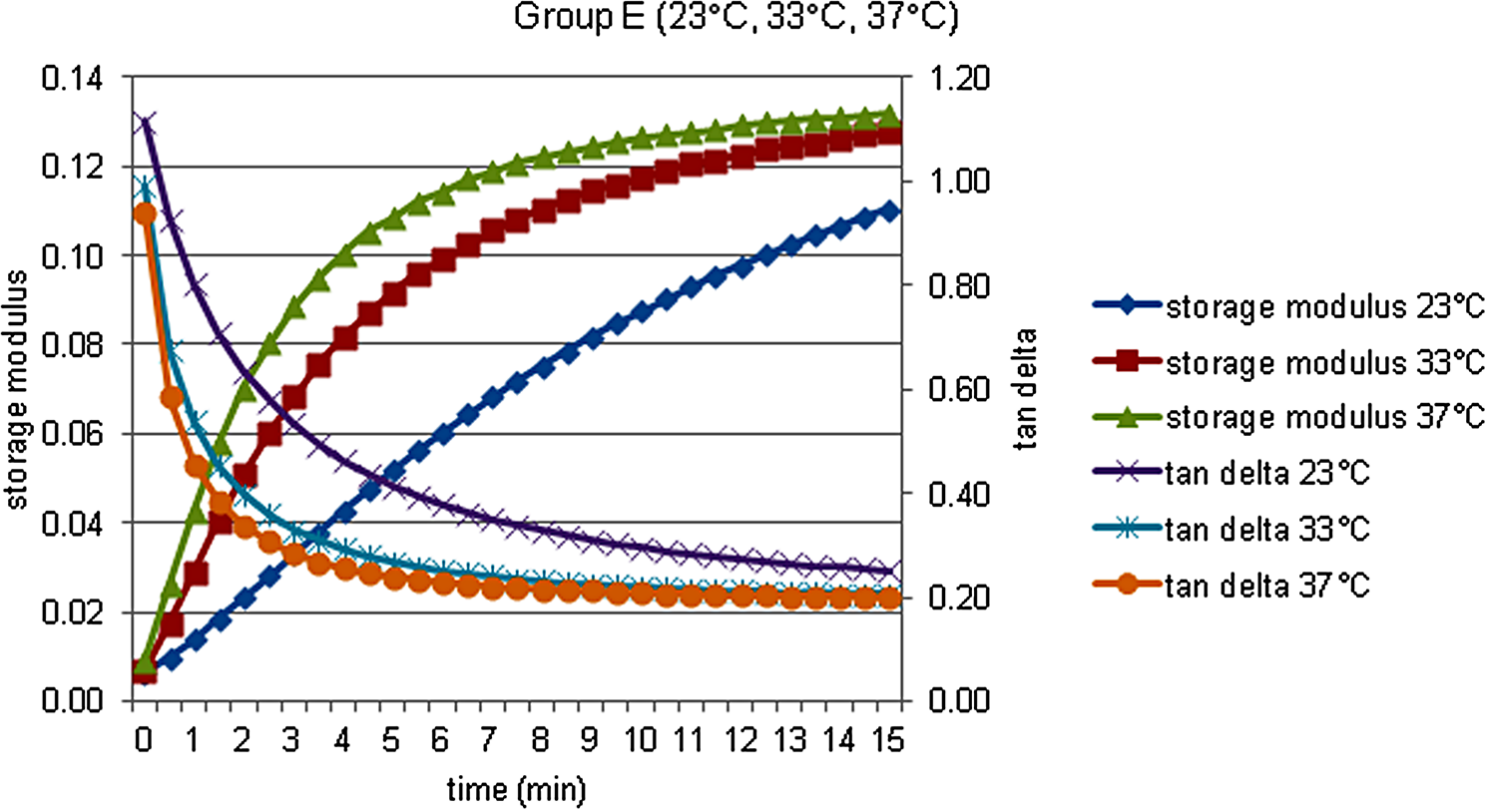
Storage modulus and tan delta against time measured at 23, 33, and 37  °C in Group E

The storage modulus (G’), the loss modulus (G’’), the loss tan delta (tan δ) and complex viscosity (η’) data of each material at 23, 33 and 37 °C, were obtained and shown in Figs. 2, 3, 4, 5 and 6.

At 0 min, there is a statistically significant difference in terms of storage modulus at different temperatures for the group C, this difference is due to the lower storage modulus at 37 °C. There is also a statistically significant difference in terms of storage modulus in group D, and storage modulus values at 33 and 37 °C are higher than at 23 °C. At the end of the 15th minute, which was determined as the time to finish the measurements and trim the lining materials, the change the storage modulus values at 33 and 37 °C was statistically significant in all groups except the group E. Within the A and B groups, the storage modulus values at 37 °C were also statistically significantly higher. For each group, statistical comparison at different times in terms of materials and temperatures was demonstrated in Fig. 2 for the group A, in Fig. 3 for the group B, in Fig. 4 for the group C, in Fig. 5 for the group D and in Fig. 6 for the group E.

**Fig. 7:**
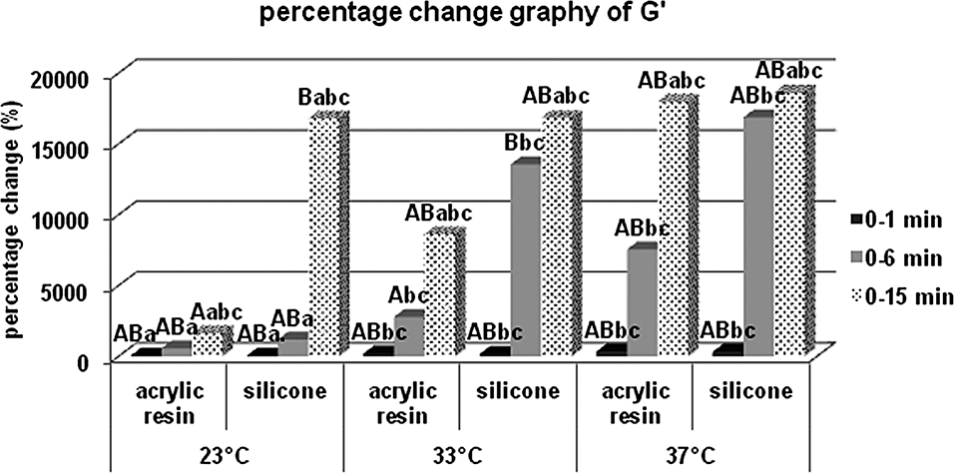
Percentage change graph of storage modulus (G’). Bars; with different uppercase letters (in the same time and temperature) and different lowercase letters (in the same time and contents) were determined to be statistically differences

**Fig. 8:**
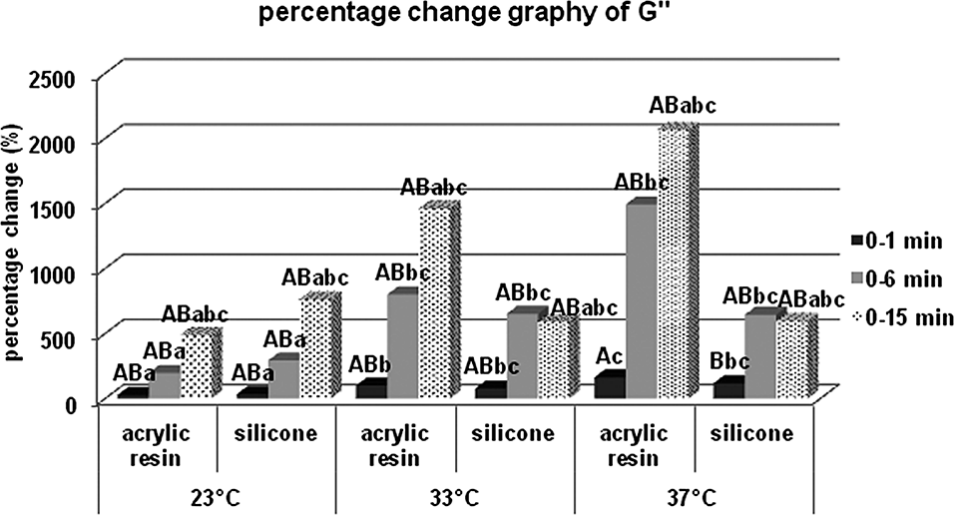
Percentage change graph of loss modulus (G’’). Bars; with different uppercase letters (in the same time and temperature) and different lowercase letters (in the same time and contents) were determined to be statistically differences

**Fig. 9:**
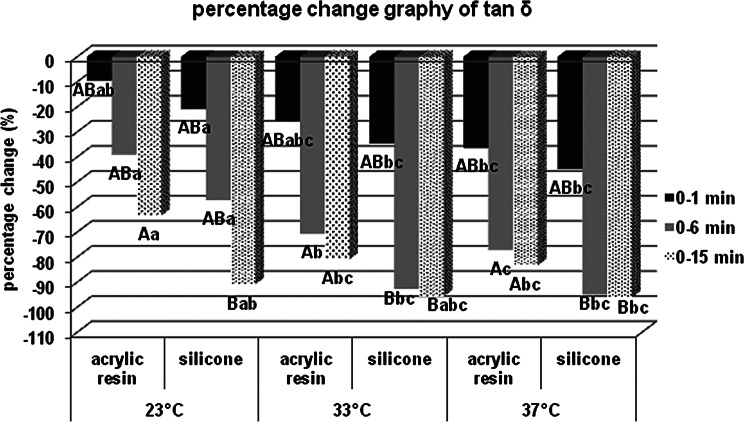
Percentage change graph of tan delta (tan δ). Bars; with different uppercase letters (in the same time and temperature) and different lowercase letters (in the same time and contents) were determined to be statistically differences

**Fig. 10:**
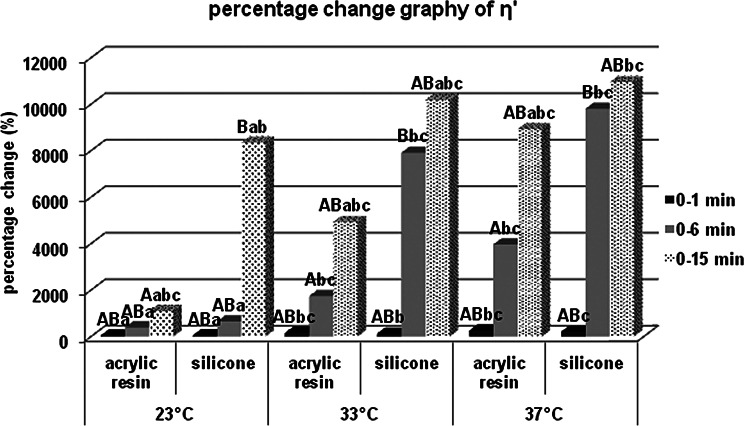
Percentage change graph of complex viscosity (η’). Bars; with different uppercase letters (in the same time and temperature) and different lowercase letters (in the same time and contents) were determined to be statistically differences

There was no statistically significant difference in terms of storage modulus values at 23, 33 and 37 °C between acrylic and silicone samples at the 0th minute, which was specified as the measurement start time, but both acrylic and silicone samples at the 6th and 15th minutes for 23 °C respectively. Storage modulus values at 33 and 37 °C are statistically significantly higher. When the percentage changes of the values according to the starting time are examined, the percentage increase in the storage modulus values at the 6th minute compared to 0. minute in the acrylic and silicone samples, respectively, compared to 23 °C; It was statistically significantly higher at 33 and 37 °C. According to Bonferroni Correction, there is no statistically significant difference between different temperatures in terms of the percentage change in the storage modulus of both acrylic and silicone samples at 0. minute or 15. minute. For each group, statistical comparison of the percentage changes of storage modulus (MPa) at different times in terms of material contents and temperatures were given in Fig. 7 for the storage modulus, in Fig. 8 for the loss modulus, in Fig. 9 for the tan delta modulus, in Fig. 10 for the complex viscosities.

## Discussion

This in vitro study evaluated the rheological properties of some soft lining materials, to compare the rheological properties of some soft lining materials and viscoelastic behaviour at different temperatures. It has been suggested that the intraoral temperature (33–35 °C) is a few degrees lower than body temperature (37 °C) when the mouth is open, and that 33 °C ± 0.5 is a suitable value for the evaluation of the properties of the impression materials in the oral environment [3]. In the present study was found that soft lining materials had different viscoelastic properties and most of the speciments showed different rheological behavior at 23 °C, 33 °C and 37 °C, therefore the null hypothesis was rejected.

The reaction rate increased from 23 °C to 33 °C and 37 °C, the greatest difference was observed between 23 °C and 37 °C, and the least difference was between 33 °C and 37 °C. This supports that the temperature affects the polymerization rate, working time and curing time [14]. For these reasons, attention should be paid to the ambient temperature during the use of soft lining materials, and the processes should be accelerated in hot environments.

In the present study, a significant difference was found between the storage (G’) and tan delta (tan δ) values of acrylic-based temporary and silicone-based permanent soft materials at the 15th minute, which was determined as the trimming time. Silicone-based soft lining materials, which have a higher storage module and significantly lower tan delta value than acrylic-based soft lining materials, are more elastic. This allows silicones to be more easily trimmed [16].

Tan delta, which is the ratio of loss modulus to storage modulus, is an important parameter in the examination of viscoelastic properties. High tan delta values indicate more energy loss and more viscous behavior, while low tan delta values indicate increased elastic properties [17, 18]. Chladek et al. [19] stated that long-term usable soft lining materials with high tan delta value, which have better ability to absorb functional stress. In the present study, it has been proven that acrylic-based temporary soft lining materials (Visco Gel -group A, Trusoft-group E) with high tan delta value are more viscous, displace more against forces and transmit the incoming forces to the supporting tissues at a lower degree.

When all groups were compared, it has been proven that Sofreliner Tough S (Group B), which is used for tissue healing, showed a lower tan delta value and was more stable with lower fluency than Visco Gel (Group A) and Trusoft (Group E). On the other hand, Ufi Gel P (Group D) showed similar results to Trusoft (Group E) in tan delta parameter, showing that although it was suitable for long-term use, it can distribute stress more than other silicone-based soft lining materials in short-term use. This should be taken into account in the choice of products to be employed.

Among the samples we used in the present study, it was found that Visco Gel (Group A) showed significantly higher fluidity than other groups, and it was more difficult to manipulate and provide sufficient thickness compared to other samples.

Murata et al. [20] stated that silicone-based soft lining materials showed significantly higher storage modulus (G’) values and lower tan delta values than acrylic-based temporary soft materials. In their functional tests with these materials, they found silicones to be more effective than temporary acrylic materials in terms of maximum bite force. The reason for this situation has been shown to be much higher elastic properties of silicones with higher storage modulus and tan delta value close to 0, and this was compatible with the results of the present study.

Ufi Gel P (Group D) gained a higher tan delta value than the silicon-based Sofreliner Tough S (Group B) and Sofreliner Tough M (Group C) and turned into a structure that can absorb more force. It was observed that the tan delta values of all groups investigated at the beginning times were higher than the last measurement times. Visco Gel (Group A) and Trusoft (Group E) have higher tan delta values than silicones, which can be attributed to the formation of the chain reaction without cross-linking due to their chemical structure.

When the rheological parameters of Visco Gel (Group A), Sofreliner Tough S (Group B) and Trusoft (Group E) samples, which were suitable for tissue healing purposes, were examined, they showed significantly lower storage modulus and complex viscosity values compared to other sample groups. Polyvinylsiloxane content and cross-linked chain structure can be shown as the reason why Sofreliner Tough S (Group B) had numerical and statistically significant differences in loss modulus and tan delta values compared to Visco Gel (Group A) and Trusoft (Group E). As stated by Lima et al. [21], the lower plasticizer concentration may have affected Trusoft (Group E)’s lower value in loss modulus and tan delta data compared to Visco Gel (Group A).

Chladek et al. [19] reported that the filler amount of the silicone-based soft material increased from 6 to 20%, increasing the complex modulus as well as reducing the loss modulus. It was postulated that the loss modulus obtained with these findings, was affected by the combined fillers and the chemical bonding between the filler and the polymer matrix. Considering the above information, the higher tan delta value of Sofreliner Tough S (Group B) than Sofreliner Tough M (Group C) can be accounted for the findings of the present study and its reduced filler content.

It was determined that all parameters were affected more especially in the first minutes in both silicone-based and acrylic-based temporary soft lining materials, and at the end of the 15th minute, no difference was observed between the percentage changes of acrylic-containing and silicone-containing groups. This percentage difference, which is clearly seen in the first 6 min, proves that the temperature affects the polymerization especially at the operating time. At the 15th minute, although there is a numerical difference between the values, since acrylic and silicone soft lining materials have reached sufficient final viscoelasticity; there is no statistically significant difference at 23, 33 and 37 °C in the percentage changes observed between the experiment start time and the experiment end time.

Considering the rheological parameters of all soft lining materials, the processes such as manipulation, intraoral shaping, trimming and polishing of silicone-based Sofreliner Tough M, which gains high elastic properties in a short time (15 min), are expected to be easier and shorter than others, while the manipulation, intraoral shaping, trimming and polishing processes of acrylic-based Visco Gel and Trusoft, which have more fluid and low elastic properties, are more difficult than others.

Although the initial rheological findings of the silicone-based Sofreliner Tough S soft lining material, which is offered to the market with both permanent and temporary and tissue healing properties, are different from the acrylic-based temporary soft lining material, in terms of the rheological information obtained during the test period, they may be preferred in order to eliminate the disadvantages of the acrylic based soft lining materials.

For dental soft polymer, especially for tissue conditioner, viscoelastic polymers with higher viscous elements than elastic ones are more suitable because they are more effective at reducing functional stress. This is because the material has strong flow properties and a high loss tan delta due to the lower storage modulus (for elastic elements) and greater loss modulus (for viscous materials) [20]. In complications such as pain, retention problems, exostosis and prosthesis dissatisfaction, it may be recommended to use high-elastic soft lining materials such as silicone-based soft lining materials. In cases where pain complaint continues and tissue trauma is very high, it can be recommended to use viscoelastic acrylic based temporary soft lining materials with lower storage modulus and higher tan delta value and high viscosity, provided that they are replaced often.

Limitations of the present study include the fact that only the rheological properties of some soft lining materials that were preferred at different temperatures were evaluated. However, physical and viscoelastic properties of soft lining materials under chewing forces were not evaluated. In addition, the long-term clinical follow-up report and randomized clinical trials and clinical use of these in vitro study results are also important. Studies are also needed that will determine how stresses caused by different occlusal forces resulting from the effects of chewing muscles affect the physical and mechanical properties of soft lining materials.

Despite the variety of rheological studies, there are many different types of mucosa and a wide range of indications. Since all the desired properties are not present in a single material, it should be tried to select the materials with the least deterioration rate in the longest period considering the elastic properties. Despite the fact that the qualities of soft lining materials have significantly improved, they still have a number of limitations. Further research needs to be performed to produce the most ideal soft lining materials.

## Conclusions

Within the limitations of this in vitro study, the following conclusions were drawn:

1. There were significant changes in the rheological parameters of all materials. Also temperature affected the initial rheological properties, and polymerization reaction of all the materials, depending on temperature increase, accelerated.
2. Graphics of the storage modulus (MPa) and the loss tan delta against time measured at 33 °C were more similar to graphics at 37 °C than at 23 °C.
3. At the end of the test (t¹5), at all the temperatures, Sofreliner Tough M had the highest storage modulus values (*P* < .05) while at all the temperatures, Visco Gel had the highest loss tan delta values (*P* < .05).
4. Silicone-based Sofreliner Tough M had high value elastic properties, while the acrylic-based Visco Gel and Trusoft had low value elastic properties.

## Data Availability

The authors declare that the data supporting the findings of this study are available within the paper.
